# Supplementary material for: Oscillatory integration windows in neurons
Source: Nat Commun. 2016 Dec 15;7:13808. doi: 10.1038/ncomms13808 (PMC5171764; doi:10.1038/ncomms13808)
Supplement: Supplementary Information — Supplementary Figure. [file ncomms13808-s1.pdf]

Gupta et al.
Supplementary Figure 1

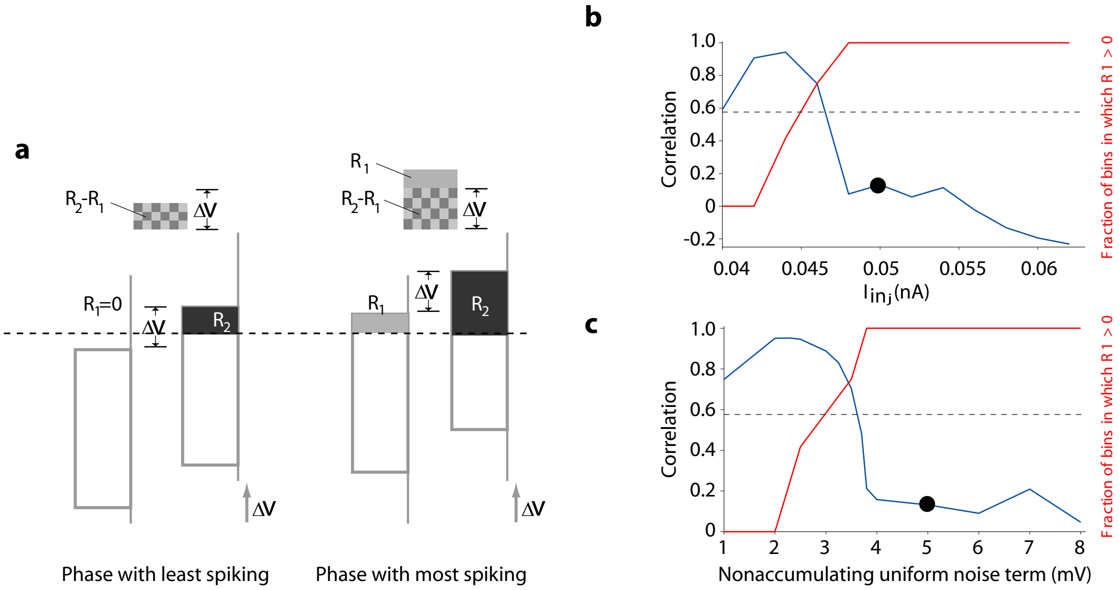

Simulations with uniform noise. (a) The computational model showed that, if R1=0 for some phases, R2-R1 can be phase-dependent even with uniform noise. (b,c) In uniform noise simulations, phase-dependent summation emerged only in parameter regimes in which R1=0 for some phases, a condition we never observed in vivo.
